# Juvenile polyposis in a SMAD4‐mutated child: A call for early surveillance

**DOI:** 10.1002/jpr3.70143

**Published:** 2026-01-21

**Authors:** Claudia Lorusso, Giuseppe Lassandro, Stefania Castellaneta, Valentina Palladino, Giovanni la Grasta, Vanessa Nadia Dargenio, Vittorio Labriola, Alberto Gaeta, Paola Giordano, Fernanda Cristofori, Ruggiero Francavilla

**Affiliations:** ^1^ Interdisciplinary Department of Medicine, Pediatric Section, Children's Hospital Giovanni XXIII University of Bari Aldo Moro Bari Italy; ^2^ Radiology Unit, Children's Hospital Giovanni XXIII University of Bari Aldo Moro Bari Italy

**Keywords:** endoscopy, hereditary hemorrhagic telangiectasia, tubular adenoma

## Abstract

We report the case of a 10‐year‐old boy with hereditary hemorrhagic telangiectasia (HHT) and a family history of SMAD4‐related juvenile polyposis syndrome (JPS), presenting with hypoferritinaemia unresponsive to oral supplementation. Endoscopic evaluation revealed multiple gastrointestinal polyps, including duodenal, gastric, and colonic lesions. Histology confirmed a tubular adenoma with low‐grade dysplasia. Genetic analysis identified a heterozygous pathogenic SMAD4 variant (c.1549_1550del), confirming the diagnosis of JPS. This case illustrates the early onset and clinical complexity of SMAD4‐associated JPS‐HHT overlap syndrome. It highlights the need for earlier, individualized screening in at‐risk children. Timely diagnosis and endoscopic monitoring can prevent severe complications, reduce the need for major surgery, and improve outcomes. Emerging therapies, such as Sirolimus, may offer additional benefit in managing polyp burden and anemia. Familial cascade testing was pivotal in identifying the mutation and guiding management. This report reinforces the importance of early personalized surveillance and a multidisciplinary approach in children with SMAD4 mutations.

## 1 INTRODUCTION

Juvenile polyposis syndrome (JPS) is a rare autosomal dominant disorder characterized by multiple hamartomatous polyps in the gastrointestinal tract and an increased risk of malignancy. Mutations in the SMAD4 gene can lead to a combined phenotype of JPS and hereditary hemorrhagic telangiectasia (HHT), complicating the clinical picture.

## 2 CASE REPORT

We report the case of a 10‐year‐old male patient with a clinical diagnosis of HHT, referred to our unit for evaluation. He had a past medical history of iron deficiency anemia and, at the time of presentation, showed low serum ferritin levels despite multiple courses of oral iron supplementation. He had no history of rectal bleeding.

**Figure 1:**
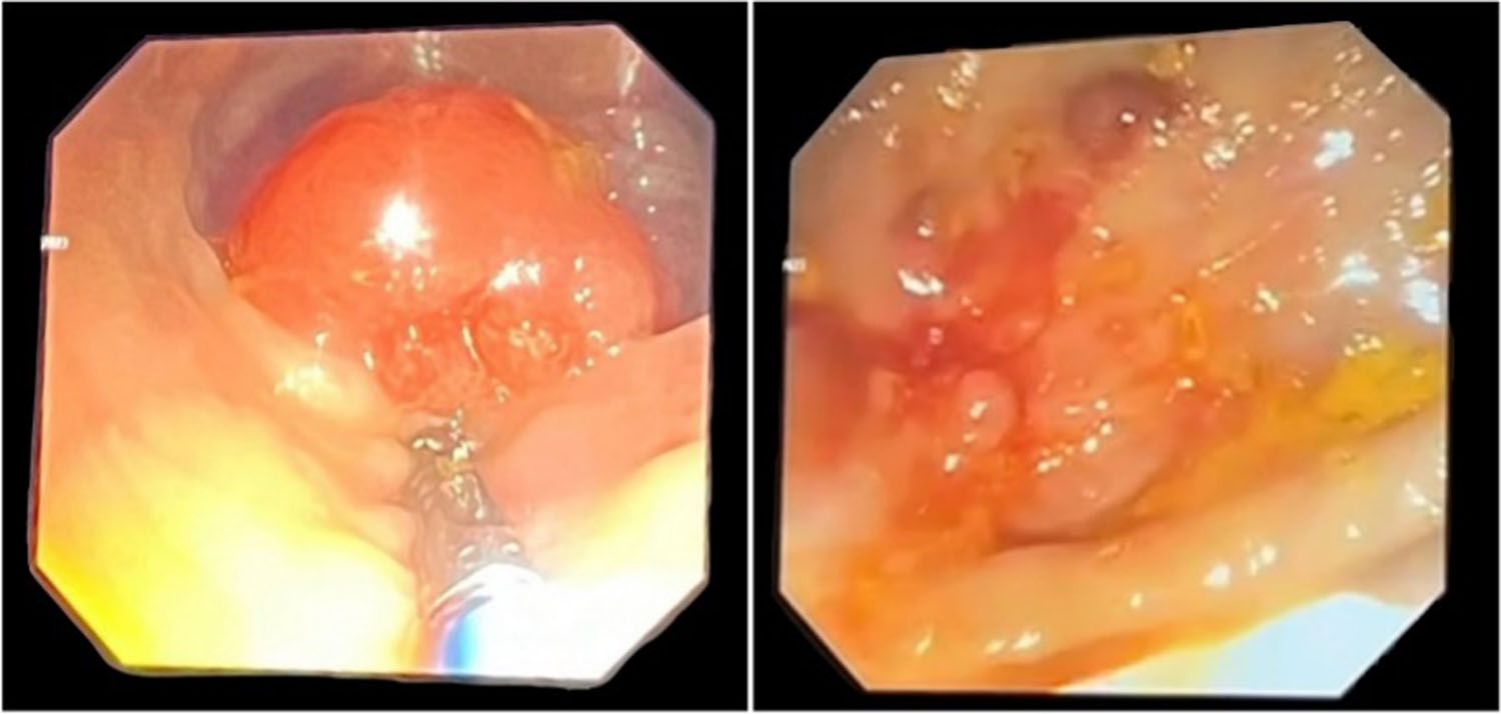
Colonic pedunculated (left) and sessile (right) polyps.

His family history was notable for a pathogenic SMAD4 gene mutation in his mother, who previously underwent total gastrectomy for gastrointestinal polyposis, and for a confirmed diagnosis of JPS in his sister. The patient's past medical history included surgically corrected interventricular septal defect with associated tricuspid valve disease, left inguinal hernia repair, and strabismus. Upon admission, laboratory tests revealed hemoglobin of 11.8 g/dL, mean corpuscular volume (MCV) of 76.18 fL, and a ferritin concentration of 8.2 ng/mL. Fecal occult blood tests were persistently positive. Esophagogastroduodenoscopy revealed a 0.4 cm polypoid lesion at the gastroesophageal junction, a 0.3 cm lesion in the gastric antrum, and multiple polyps (0.2–0.4 cm) in the second portion of the duodenum. Colonoscopy demonstrated pedunculated and sessile polyps throughout the colon, ranging in size from 0.5 to 2 cm (Figure 1). Histopathological examination of a colonic polyp confirmed the presence of a tubular adenoma with low‐grade dysplasia. Molecular genetic analysis identified a heterozygous frameshift variant in the SMAD4 gene (c.1549_1550del; p. Ser517Hisfs*9), classified as likely pathogenic and of germline origin. Based on the Jass diagnostic criteria, a definitive diagnosis of JPS was established. The patient was subsequently enrolled in a structured program of endoscopic surveillance.

## 3 DISCUSSION

The present case of a 10‐year‐old boy with a germline SMAD4 mutation and overlapping features of JPS and HHT highlights the urgent need to implement early and systematic endoscopic surveillance protocols in pediatric populations at risk. JPS is a rare but clinically significant hamartomatous polyposis disorder with a well‐documented predisposition to gastrointestinal malignancies, especially in individuals harboring SMAD4 mutations. Timely recognition, genetic diagnosis, and proactive endoscopic monitoring are critical to preventing life‐threatening complications, minimizing surgical interventions, and improving long‐term outcomes.^1^, ^2^

JPS has an estimated incidence of 1 in 100,000–160,000 individuals and is clinically defined by the development of multiple hamartomatous polyps, most commonly affecting the colon, rectum, stomach, and small intestine.^1^ Although histologically benign, these lesions are precancerous and carry a significant risk of adenomatous transformation and subsequent carcinoma.^2^, ^3^ This malignant potential is particularly heightened in patients with SMAD4 mutations due to impaired transforming growth factor (TGF)‐signaling, which disrupts normal control of epithelial proliferation, apoptosis, and tissue homeostasis.^2^, ^3^

The case under discussion reflects a growing body of literature documenting the phenotypic complexity of SMAD4‐related JPS.^4^ This patient presented with iron‐deficiency anemia unresponsive to oral supplementation, multiple gastrointestinal polyps, and a pathogenic SMAD4 variant, alongside evidence of HHT. These overlapping features, often referred to as a “combined syndrome,” are now well recognized in SMAD4 mutation carriers and illustrate the multisystemic burden of disease that can begin in early childhood.^5^

Several studies have highlighted the significant morbidity linked to delayed diagnosis in SMAD4‐positive patients. Gonzalez et al.^6^ reported early GI symptoms, frequent major surgeries, and even adolescent‐onset carcinoma, emphasizing the subtle and variable pediatric presentation. As SMAD4 mutations may not meet full HHT criteria, genetic testing remains essential, especially in cases with suggestive history or unexplained anemia.^6^

The European Society for Paediatric Gastroenterology, Hepatology and Nutrition (ESPGHAN) 2019 guidelines recommend initiating genetic screening and colonoscopic surveillance between the ages of 12 and 15 in asymptomatic individuals at risk for JPS, with the option to begin earlier if clinical features such as anemia or gastrointestinal bleeding are present.^7^ This case reinforces the importance of applying these criteria rigorously in clinical practice to ensure timely diagnosis and intervention. Indeed, early adenomatous changes and clinically significant anemia are increasingly reported before the second decade of life, particularly in SMAD4‐related disease.^6^ Therefore, this case supports a revision of surveillance strategies, advocating for individualized earlier endoscopic assessment based on clinical presentation, family history, and genetic findings.

The importance of early detection and intervention cannot be overstated. Endoscopic resection of polyps in early stages can not only alleviate symptoms such as bleeding and anemia but also preclude the need for radical surgeries, which carry their own long‐term morbidity. Moreover, surveillance offers an opportunity to histologically assess the nature of the polyps, track progression, and tailor follow‐up intervals accordingly. After initial polyp clearance, colonoscopic follow‐up every 1–5 years is recommended, with adjustments based on polyp burden and histopathology.^7^ Upper gastrointestinal surveillance is indicated in anemic or symptomatic individuals, with small bowel imaging reserved for selected cases using modalities such as computed tomography enterography, capsule endoscopy, or balloon‐assisted enteroscopy.^7^

The therapeutic landscape for JPS is also evolving, particularly with the off‐label use of mTOR inhibitors such as Sirolimus. As demonstrated by Martin‐Masot et al.,^8^ Sirolimus has shown promise in reducing polyp burden and controlling anemia in SMAD4‐mutated patients. In that study, an 8‐year‐old child with refractory anemia experienced marked improvement after treatment. By modulating the mammalian target of rapamycin (mTOR) pathway, intersecting with TGF‐and Phosphatase and TENsin homolog (PTEN)/Bone Morphogenetic Protein Receptor, type 1A (BMPR1A) signaling, Sirolimus indirectly downregulates SMAD4‐driven proliferative signals and may delay dysplastic progression.

Although sirolimus therapy was not indicated in this case, it may represent a valuable option in patients with refractory polyp burden, persistent anemia despite iron supplementation, small bowel involvement, or hypoalbuminaemia.

Equally important is the integration of genetic counseling and family‐based risk assessment into the management of JPS. In the present case, the identification of a SMAD4 mutation in the patient was made possible through familial screening, as his mother and sister had already been diagnosed with related conditions. This underscores the value of cascade testing in at‐risk relatives and the importance of comprehensive pedigree analysis in guiding early intervention. Given the variable expressivity and incomplete penetrance observed in SMAD4 mutation carriers, close monitoring of asymptomatic individuals remains essential.^6^

## 4 CONCLUSION

In conclusion, this case reinforces the need for early and systematic endoscopic surveillance in children with or at risk for SMAD4‐related JPS. Initiating screening at a young age enables timely detection and resection of polyps, thereby reducing the cumulative risk of gastrointestinal carcinoma, limiting the need for extensive surgical interventions. As knowledge of the genetic and clinical variability of JPS expands, surveillance should follow a personalized approach based on genotype, symptoms, and family history. Multidisciplinary collaboration remains key to delivering comprehensive care for these complex pediatric cases.

## CONFLICT OF INTEREST STATEMENT

The authors declare no conflicts of Interest.

## ETHICS STATEMENT

Informed patient consent was obtained for publication of the case details.
